# Sexually dimorphic gene expression emerges with embryonic genome activation and is dynamic throughout development

**DOI:** 10.1186/s12864-015-1506-4

**Published:** 2015-04-14

**Authors:** Robert Lowe, Carolina Gemma, Vardhman K Rakyan, Michelle L Holland

**Affiliations:** Centre for Genomics and Child Health, Blizard Institute, Barts and The London School of Medicine and Dentistry, 4 Newark Street, London, E1 2AT UK

**Keywords:** Sexual dimorphism, Pre-implantation embryo, Chromatin, Gene expression, Epigenetics, Development, Mouse, Mammalian, Sex-specific

## Abstract

**Background:**

As sex determines mammalian development, understanding the nature and developmental dynamics of the sexually dimorphic transcriptome is important. To explore this, we generated 76 genome-wide RNA-seq profiles from mouse eight-cell embryos, late gestation and adult livers, together with 4 ground-state pluripotent embryonic (ES) cell lines from which we generated both RNA-seq and multiple ChIP-seq profiles. We complemented this with previously published data to yield 5 snap-shots of pre-implantation development, late-gestation placenta and somatic tissue and multiple adult tissues for integrative analysis.

**Results:**

We define a high-confidence sex-dimorphic signature of 69 genes in eight-cell embryos. Sex-chromosome-linked components of this signature are largely conserved throughout pre-implantation development and in ES cells, whilst the autosomal component is more dynamic. Sex-biased gene expression is reflected by enrichment for activating and repressive histone modifications. The eight-cell signature is largely non-overlapping with that defined from fetal liver, neither was it correlated with adult liver or other tissues analysed. The number of sex-dimorphic genes increases throughout development. We identified many more dimorphic genes in adult compared to fetal liver. However, approximately two thirds of the dimorphic genes identified in fetal liver were also dimorphic in adult liver. Sex-biased expression differences unique to adult liver were enriched for growth hormone-responsiveness. Sexually dimorphic gene expression in pre-implantation development is driven by sex-chromosome based transcription, whilst later development is characterised by sex dimorphic autosomal transcription.

**Conclusion:**

This systematic study identifies three distinct phases of sex dimorphism throughout mouse development, and has significant implications for understanding the developmental origins of sex-specific phenotypes and disease in mammals.

**Electronic supplementary material:**

The online version of this article (doi:10.1186/s12864-015-1506-4) contains supplementary material, which is available to authorized users.

## Background

Sex determines anatomical, physiological and behavioural development in mammals. This developmental divergence arises as a consequence of sex-chromosome complement and is largely, although, not exclusively mediated through the organisational and activational effects of sex-specific hormones [1]. In adulthood, sex-specific gene expression is widespread in somatic tissues [2]. Consequentially, sex influences a plethora of complex traits that do not directly relate to reproductive roles. Exemplifying this, many diseases exhibit sex bias in prevalence or severity [3], and association of genetic variants with disease states is sex-dependent [4,5].

The ‘Four Core Genotypes’ mouse model has been used to dissect the contribution of sex-chromosome complement and the organisational and activational effects of sex-specific hormones [6], revealing that both hormones and chromosomal complement exert independent and divergent effects over metabolic and behavioural phenotypes [7,8]. Furthermore, animal models of the Developmental Origins of Health and Disease hypothesis reveal differential outcomes of early environmental insults dependent on sex [9], suggesting that sex should be considered as third parameter in any gene-environment interactions. Despite this, the origin and nature of these sex based differences are largely unexplored.

There is evidence that sexually dimorphic gene expression arises prior to gonadal differentiation, even in pre-implantation blastocysts, with both sex-linked and autosomal genes affected [10,11]. This dimorphism could determine the effects of specific environmental factors on long term developmental outcomes. Indeed, embryo culture medium induces long-term effects on glucose homeostasis in a sexually dimorphic manner [12]. Despite the relevance to human health and reproductive technologies, the origins and developmental dynamics of mammalian sexual dimorphism have not been characterized in detail. Here we report a high confidence signature of sexually dimorphic genes in the mouse coincident with embryonic genome activation using unmanipulated embryos, and from fetal and adult liver and performed comparative analyses using data from other sources. Our findings demonstrate that dimorphic expression emerges in the early cleavage embryo and is highly dynamic throughout development. Furthermore, we show that this is reflected in the chromatin structure. Through defining the nature and developmental origins of sexual dimorphism we provide a background for interpreting gene-environment interactions in directing developmental outcomes.

## Results

### Sexually dimorphic gene expression emerges very early in development

**Figure 1:**
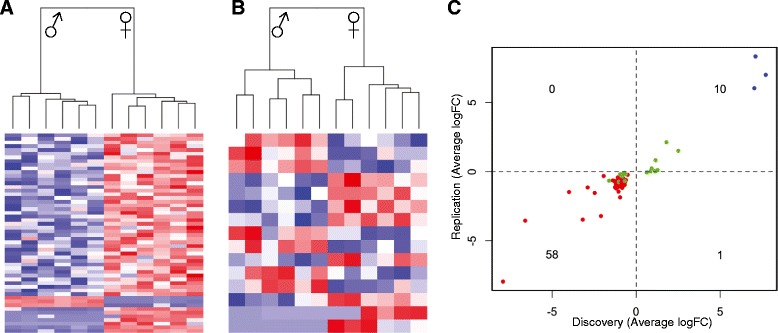
Identification and biological replication of sexually dimorphic genes identified in eight-cell embryos. Transcripts were defined as sex dimorphic from RNA-seq data using a genome-wide corrected p-value < 0.1 and −0.5 < log fold change (male/female) > 0.5 (**A**) Sexually dimorphic X-linked (51) and Y-linked (3) genes identified in the discovery set of six male and six female eight-cell embryos. (**B**) Autosomal genes (15) identified in the discovery set of six male and six female eight-cell embryos. Transcripts more highly expressed in a particular sex are indicated in red, those less expressed are indicated in blue. Rows representing individual transcripts were scaled to mean = 0, standard deviation = 1 (**C**) Average log fold change (male/female) for the discovery set (x-axis) plotted against log fold change (male/female) for an independent set of eight female and four male embryos (r = 0.95, p-value < 2.2 × 10^−16^). X-linked genes are indicated in red, Y-linked genes are indicated in blue and autosomal genes are indicated in green.

Eight-cell embryos provide the first snap-shot in mice after the initiation of embryonic genome activation at the 2 cell stage, when maternal transcripts have been depleted [13,14]. We isolated 12 eight-cell embryos for a discovery set and a further 12 for validation. Importantly, the embryos were produced by natural mating of inbred C57BL/6 J mice, without super-ovulation or *in vitro* culture, thereby providing a unique opportunity to examine sexually dimorphic expression *in vivo*. We generated transcriptome profiles for individual embryos using an adapted single-cell RNA-seq protocol (sequencing statistics are given in Additional file 1: Table S1). To our knowledge, this is the largest number of genome-wide transcriptome profiles from individual mouse embryos to date. We detected 13 469 transcripts expressed across all eight cell embryos, which is greater than previous microarrays of blastocysts, but less than other RNA-seq data from eight cell embryos that was sequenced at greater depth [11,14]. The sex of the embryos was determined by plotting expression of the Y chromosome gene, *Eif2s3y* and the X chromosome gene, *Xist*, as both are expressed in a sex-specific manner in murine blastocysts [10]. A clear separation of 2 distinct groups consisting of 6 female and 6 male embryos for the discovery set was found (Additional file 2: Figure S1). We then sought to identify genes that were differentially expressed between male and female embryos, revealing 69 genes with a genome-wide corrected p-value < 0.1, (Benjamini-Hochberg correction) and −0.5 < log_2_ (male/female) > 0.5 (Table 1). The majority of sex dimorphic differences (78%) originated from the X and Y chromosomes (51/69 and 3/69, respectively; Figure 1A), whilst ~22% (15/69) were autosomal in origin (Figure 1B). All X-linked genes were more expressed in female eight-cell embryos. Sex-dimorphic autosomal genes were equally distributed amongst the sexes.

**Table 1:** **Genes defined as sexually dimorphic from eight-cell embryos**

**Gene name**	**CHR**	**Start**	**End**	**Strand**	**baseMean**	**log2FoldChange**
Xist	chrX	103460373	103483233	-	275.66	−9.01
Ube1y1	chrY	818713	844224	+	232.95	9.02
Ddx3y	chrY	1260715	1286613	-	153.51	8.49
Bex1	chrX	136213972	136215513	-	168.51	−6.46
B230206F22Rik	chrX	103560910	103623754	-	34.38	−6.73
Sms	chrX	157443954	157492046	-	6792.54	−1.32
Eif2s3y	chrY	1010612	1028598	+	224.03	5.94
Rbm3	chrX	8138975	8145802	-	3756.52	−1.06
Fthl17	chrX	9033486	9034333	+	18.80	−5.67
Huwe1	chrX	151803282	151935417	+	2245.04	−0.81
Hprt	chrX	52988078	53021660	+	16923.45	−1.10
Eif2s3x	chrX	94188709	94212651	-	3306.76	−0.81
Syap1	chrX	162856843	162888462	-	2009.79	−1.13
Yy2	chrX	157566119	157568985	-	237.80	−2.07
Eif1ax	chrX	159372195	159385699	+	1826.30	−1.01
Ccng1	chr11	40748552	40755286	-	403.37	1.88
D7Ertd715e	chr7	59969577	59974431	-	365.59	−1.63
Atp6ap2	chrX	12587759	12617051	+	1103.98	−1.02
Uba1	chrX	20658421	20683179	+	4718.65	−0.82
Ddx3x	chrX	13281022	13293983	+	4503.61	−0.96
Timm8a1	chrX	134537258	134541629	-	3804.40	−1.20
Tsr2	chrX	151087094	151096543	-	2495.42	−0.76
Med14	chrX	12675371	12761973	-	725.57	−1.36
Usp38	chr8	80980733	81014906	-	1115.47	−0.99
Rhox5	chrX	37754608	37808878	+	27.14	−3.92
Gnl3l	chrX	150983133	151017322	-	910.96	−0.74
Kdm6a	chrX	18162667	18279358	+	481.77	−0.98
Als2cr11	chr1	59050506	59094900	-	9.24	4.02
Aen	chr7	78895927	78908833	+	513.62	0.99
Bcap31	chrX	73686183	73716175	-	1554.31	−0.63
Rbbp7	chrX	162760372	162779090	+	4036.08	−0.81
Acsl4	chrX	142317993	142390535	-	1262.01	−1.08
Idh3g	chrX	73778963	73786897	-	2177.00	−0.71
Lamp2	chrX	38405064	38456455	-	1278.91	−1.03
Rbmx	chrX	57383348	57393036	-	321.01	−0.91
Porcn	chrX	8193850	8206525	-	303.00	−1.11
Ssr4	chrX	73787028	73790828	+	798.15	−0.82
Otud5	chrX	7841831	7874858	+	728.02	−0.71
1810030O07Rik	chrX	12654884	12673546	-	134.89	−1.85
Atrx	chrX	105797615	105929372	-	707.08	−0.85
Prps1	chrX	140456603	140476140	+	8178.23	−0.77
Aifm1	chrX	48474944	48513426	-	387.98	−1.18
Gm14511	chrX	8975712	8976559	-	31.79	−3.30
Mapkapk2	chr1	131053704	131097543	-	46.62	2.72
Wdr45	chrX	7722249	7728201	+	2311.35	−0.85
Cox7b	chrX	106015700	106022450	+	116.54	−1.37
Mad2l1bp	chr17	46147385	46153551	-	1191.38	−0.92
Tmem56	chr3	121202010	121263316	-	542.41	−1.12
Rpl10	chrX	74270816	74273135	+	14774.94	−0.80
Cybasc3	chr19	10577723	10589830	+	465.93	0.85
Mtf1	chr4	124802549	124849800	+	512.08	−0.75
Cks2	chr13	51645232	51650662	+	2503.02	−0.66
Gm14458	chrX	8985900	8986755	-	5.13	−3.53
Cdca7	chr2	72476219	72486890	+	867.49	0.67
Pgrmc1	chrX	36598225	36606079	+	414.68	−1.00
Thoc2	chrX	41794994	41911901	-	381.21	−1.06
Cited1	chrX	102247440	102251769	-	3344.44	−0.53
Ypel5	chr17	72836704	72851195	+	2403.16	−0.70
Cdk16	chrX	20688493	20699877	+	802.78	−0.70
Dnajc28	chr16	91614257	91618999	-	10.24	3.47
Fmr1	chrX	68678555	68717961	+	587.22	−1.20
Gla	chrX	134588169	134601005	-	476.84	−1.06
Hnrnph2	chrX	134601286	134607054	+	879.15	−0.74
Mpp1	chrX	75109733	75130949	-	430.95	−1.07
Msl3	chrX	168654118	168673902	-	355.99	−1.12
Tbc1d25	chrX	8154472	8176181	-	258.99	−1.15
Trappc1	chr11	69323986	69325793	+	118.07	1.26
Utp14a	chrX	48256934	48282449	+	619.81	−0.73
Xiap	chrX	42067836	42109664	+	449.57	−0.86

Dimorphic genes were defined from RNA-seq data generated from 6 male and 6 female eight-cell embryos (adjusted p-value < 0.1, (Benjamini-Hochberg correction) and −0.5 < log_2_(male/female) > 0.5). TSS = transcriptional start site.

Substantial variation between individual embryos is apparent (Figures 1A,B, Additional file 2: Figure S2), and may reflect small differences in the timing of embryonic genome activation. However, we validated sex-biased expression of these genes in another 12 eight-cell embryos isolated in an independent experiment (8 female, 4 male) (Figure 1C). Biological replicates showed a strong directional correlation (ρ = 0.95, p-value < 2.2x10^−16^), with all but one of the 69 genes showing the same sex-based expression bias, even though transcripts show average log(male/female) values small in magnitude. Three non-coding transcripts were included in the eight-cell signature, consistent with a role for non-coding RNAs in early embryo development [15]. Two of these were involved in X inactivation, *Xist* and *B230206F22Rik* (also known as *Ftx*) [16,17]. The third, *D7Ertd715e* is located immediately 3′ to the *Snrpn*/*Snurf* imprinted cluster on chromosome 7, but its function is unknown. We performed KEGG analysis of X-linked genes, using detected X chromosome genes as background and separately for the autosomal genes, using all detected autosomal genes as background. Neither analysis revealed enrichment after Bonferroni correction (p < 0.05).

### The dynamics of sexual dimorphism at different developmental stages

Having defined a high confidence, replicated gene expression signature from eight-cell embryos, we explored the temporal dynamics of these genes with respect to sex in pre- and post-implantation development. We obtained publically available transcriptome data derived from multiple stages (late two-, four- and sixteen-cell) of mouse pre-implantation development [14]. Not all embryonic stages profiled by Deng *et al.*, were included because sex could not be determined (earlier than late two-cell), only one sex was sampled, or the sex ratio was highly skewed (e.g. blastocysts). Several factors make using this data for defining stage-specific signatures problematic. The embryos profiled by Deng *et al.* are F1 hybrids of two genetically diverse inbred mouse strains (CAST/EiJ females × C57BL/6 J males). As such, embryo sex is directly confounded by genotype. They were also produced using super-ovulation, which may influence embryo development [18]. Furthermore, only small numbers of embryos were profiled at each stage (<5 total, Table 2), reducing the confidence of signatures defined *de novo*. Nonetheless, the data is useful for comparative analyses using the high confidence signature we defined from eight-cell embryos. To ensure that the coverage and dynamic range between these datasets is comparable, as we used different RNA-seq protocols, we first compared our eight-cell male embryos (n = 6) to eight-cell male embryos profiled by Deng *et al*., (n = 4), establishing a high degree of correlation between these profiles (Additional file 2: Figure S3, ρ =0.84, p-value < 2.2 × 10^−16^). To get a more expansive view of the developmental dynamics of dimorphic expression, we additionally generated embryonic stem (ES) cells derived from eight-cell embryos (2 male, 2 female) using the 2i method [19]. The 2i methodology results in ES cell lines that are transcriptionally and epigenetically similar to pre-implantation epiblast [20-22]. This was confirmed by a strong correlation of the 2i ESC transcriptome to recently published inner cell mass single-cell profiles (Additional file 2: Figure S4, ρ = 0.70, p-value < 2.2 × 10^−16^) [23]. To profile post-implantation development we generated RNA-seq profiles from 17.5 dpc (days post-coital) fetal and adult liver (12 male, 12 female at each stage). We selected liver, because sex-based differences are well characterised and prolific in adults [24]. The ES cell, fetal and adult transcriptomes were all from a C57BL/6 J background.

**Table 2:** **Sample characteristics and sources for comparative analyses**

**Sample type**	**Number of males**	**Number of females**	**Data type**	**Source**
2-cell embryos	2	3	Single cell RNA-seq	[14]
4-cell embryos	1	3	Single cell RNA-seq	[14]
8-cell embryos	5	0	Single cell RNA-seq	[14]
8-cell embryos (discovery)	6	6	Whole embryo RNA-seq	This study
8-cell embryos (replication)	4	8	Whole embryo RNA-seq	This study
16-cell embryos	2	2	Single cell RNA-seq	[14]
2i ES cells	2	2	RNA-seq	This study
2i ES cells	2	2	ChIP-seq	This study
Fetal liver	12	12	RNA-seq	This study
Placenta	2	4	Microarray	[28]
Adult liver	12	12	RNA-seq	This study
Adult liver	165	169	Microarray	[24]
Adult adipose	165	169	Microarray	[24]
Adult brain	165	169	Microarray	[24]
Adult muscle	165	169	Microarray	[24]

**Figure 2:**
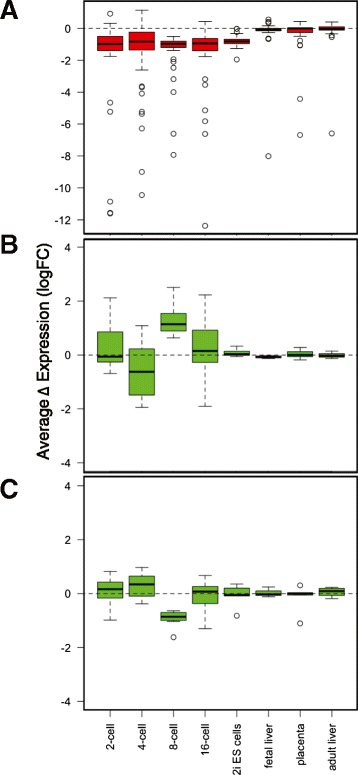
Sex biased expression of sexually dimorphic genes defined in eight-cell embryos across pre-implantation development, fetal and adult tissues. Sex-biased expression is represented as log fold change (male/female). Box plots represent mean with interquartile range. Whiskers are defined by extremes within 1.5X the interquartile range and additional points outside this range are shown as circles. (**A**) X-linked genes (red). Data for the placenta is restricted to 45/51 transcripts for which data was available (**B**) Autosomal genes (green) more expressed in male embryos. Data for the placenta is restricted to 7/8 transcripts for which data was available (**C**) Autosomal genes (green) more expressed in female embryos. Data for the placenta is restricted to 6/7 transcripts for which data was available.

Taking the two-, four- and sixteen-cell embryo data generated by Deng *et al*., together with our ES cell, fetal and adult liver data, we next examined whether the dimorphic expression of the 69 genes defined in eight-cell embryos is conserved throughout development. Unfortunately, Deng *et al*., only sampled males at the eight-cell embryo stage, precluding a direct comparison at this developmental time-point. Of the 69 signature genes, only one gene located on the Y chromosome (*Eif2s3y*) was expressed in a sexually dimorphic manner (logFC > 0.5) across all profiles, from 2 cell embryos onwards. From the four-cell stage onwards, a substantial proportion of transcripts are of embryonic origin [13,14] and the sexually dimorphic expression of the other Y chromosome genes (*Ddx3y, Ube1y1*) is established. The sex-specific expression of the majority of X chromosome genes is established from the two- to four-cell stage and maintained throughout pre-implantation development and in the ES cells (Figure 2A). However, the majority of the sex-chromosome genes within this signature do not show dimorphic expression in fetal or adult liver.

Interestingly, the 15 autosomal genes that are sexually dimorphic in eight-cell embryos did not show consistent directional changes at other time-points in pre-implantation development. As these genes were expressed at comparable levels to the sex-chromosome encoded transcripts, it seems unlikely that an increased signal to noise ratio is responsible for this. Despite not showing dimorphism at other pre-implantation stages, 14/15 of these genes show biological validation at the eight-cell stage (Figure 1C), suggesting that this stage-specific autosomal sexual dimorphism is *bona fide*. The expression level of these genes seems to be very dynamic throughout pre-implantation development (Additional file 2: Figure S5). Similar pre-implantation stage-specific expression has also been shown for genes identified as dimorphic in bovine blastocysts [11]. Indeed, global autosomal gene expression reaches a nadir at the eight-cell stage (Additional file 2: Figure S6). Collectively, we show that sex-chromosome genes show largely consistent sexual dimorphism throughout pre-implantation development and in ground state pluripotent stem cells, regardless of genetic background, but that dimorphism of autosomal genes is more temporally dynamic.

### X-linked dimorphic genes escape paternal X inactivation in pre-implantation development

The emergence of sexually dimorphic expression of *Xist* at the four-cell stage coincides with the initiation of transcriptional inactivation of the paternal X chromosome in mice [25]. Higher X-linked expression in females must therefore reflect either an up-regulation of these genes from the maternal X chromosome in females, or escape from paternal X chromosome inactivation in the pre-implantation stages. Consistent with the escape from silencing of the paternal X we find that the majority of X-linked sex dimorphic genes are located distally from the X inactivation centre (Additional file 2: Figure S7), confirming previous observations [26]. Indeed, allelic data generated by Deng *et al*., confirms that 35/38 (~92%) genes that had adequate allele-specific information were expressed from both maternal and paternal X chromosomes at one or more developmental time-points (four-, sixteen- or early blastocyst) [14]. Although there is some suggestion that inter-specific crosses may have altered X inactivation, reassuringly, two of the three transcripts solely of paternal origin are known to be involved in silencing the chromosome from which they are transcribed, *Xist* and *B230206F22Rik* (also known as *Ftx*) [16,17]. The other transcript (*Gla*) showed paternal-specific expression at the four-cell stage, with allelic data for later stages not available. Notably, silencing of paternal expression of this transcript has previously been shown to be initiated after the eight-cell and completed only by the blastocyst stage [27].

Sex-biased expression of most X chromosome genes that are dimorphic in eight-cell embryos, with the exception of *Xist,* and *Eif2s3x* is erased (−0.5 < logFC > 0.5) in fetal and adult liver (Figure 2A), but female-biased expression was retained for 5/51 X-linked genes in publically available gene expression array data from late gestation placenta [28], at log(male/female) < −0.5. As extra-embryonic tissues in the mouse demonstrate imprinted inactivation of the paternal X chromosome, we asked if dimorphic X-linked expression is more similar in general between these two tissues. Using a cut-off of log(male/female) < −0.5, we identified 79 X-linked genes showing female biased expression in placenta and found that there was a trend towards female-biased expression of these genes in the eight-cell embryos (t test, mean = −0.14, p-value = 0.046). Consistent with re-activation of the inactive paternal X chromosome in the epiblast-like 2i ES cells, the female bias in expression of the dimorphic X-linked genes identified from eight-cell embryos is maintained and expanded upon (Additional file 2: Figure S8). In fetal and adult somatic tissues, a different set of X chromosome genes are dimorphic.

### Sex-biased expression is correlated with post-translational histone modifications

Specific post-translational histone modifications are associated with particular transcriptional states and genomic features. We sought to explore the relationship of sexually dimorphic expression in pre-implantation development with chromatin features. To address this, we generated genome-wide profiles for three post-translational histone modifications in our 2i ES cells using chromatin immunoprecipitation combined with deep sequencing (ChIP-seq). 12–20 million mapped reads were generated for each mark (Additional file 1: Table S1). Determining average profiles for the 2000 most high- or low-expressed genes confirmed the expected enrichment of H3K4me3 at active gene promoters, whilst H3K27me3 was depleted (Additional file 2: Figure S9). Both H3K27me3 and H3K9me3 were enriched at repetitive elements and H3K9me3 was associated with imprinted genes, confirming the distribution of these modifications is typical of ground state pluripotency [20]. Genes located on the X chromosome conform to genome-wide distribution patterns.

**Figure 3:**
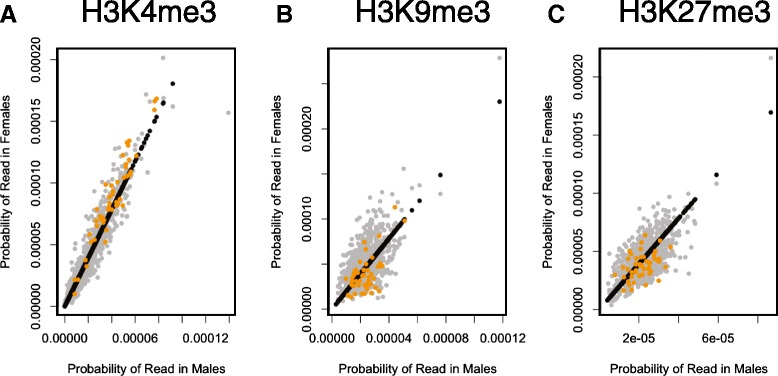
X-linked genes more expressed in female eight-cell embryos show female-biased enrichment for H3K4me3 and depletion of H3K9me3 and H3K27me3 in 2i embryonic stem (ES) cells. After correcting for sex differences in X chromosome dosage, X-linked genes that do not show sex-biased enrichment of post-translational histone modifications should fit the model (black line). All X-chromosome genes were used as background (grey points). Enrichment was calculated for the 3 kb surrounding the transcriptional start site from ChIP-seq data generated from 2i ES cells. Dimorphic X-linked genes identified from eight-cell embryos are more expressed in females (orange points). (**A**) H3K4me3 is relatively enriched in females for the dimorphic genes (FE_female_ = 1.47; p-value = 0.002) (**B**) H3K9me3 is relatively depleted in females for the dimorphic genes (FE_female_ = 0.72; p-value = 0.028) (**C**) H3K27me3 is relatively depleted in dimorphic genes (FE_female_ = 0.63; p-value = 0.005). Significance for fold enrichment was calculated using permutation tests.

Given the association of H3K4me3 with transcriptional activity, we next asked whether the X-linked genes identified as sex-dimorphic in the eight-cell embryos are more enriched for H3K4me3 in female 2i ES cells. Dimorphic expression of 46/51 of these genes is conserved in the ES cells. Before assessing relative enrichment for X-linked dimorphic genes, we first needed to account for the difference in chromosome dosage between the sexes. When ChIP-seq reads were counted in 3 kb windows surrounding the TSS, we were able to show that all three histone marks show relative enrichment in females, as expected. We developed a model allowing us to correct for chromosome dosage in subsequent analyses (see Methods). Interestingly, after applying the model for dosage correction, we found that sex-dimorphic genes show female-biased enrichment of H3K4me3 surrounding the TSS (FE_female_ = 1.47; p-value = 0.002), whilst H3K9me3 (FE_female_ = 0.72; p-value = 0.028) and H3K27me3 (FE_female_ = 0.63; p-value = 0.005) show greater enrichment in the males (Figure 3). This is consistent with enrichment of H3K4me3 in female compared to male ES cells at the X-linked *Rhox6/9* genes which is lost upon differentiation and loss of expression [29].

**Figure 4:**
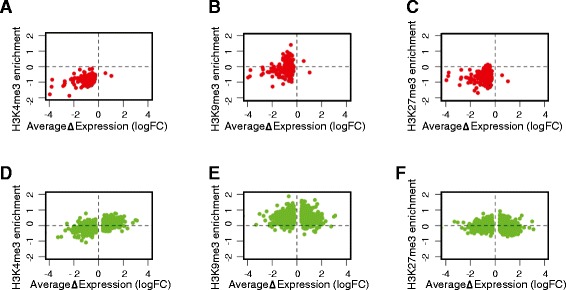
Histone post-translational modifications associated with the transcriptional start site (TSS) of sexually dimorphic genes in 2i embryonic stem cells. Transcripts were defined as sex dimorphic from RNA-seq data using a genome-wide corrected p-value < 0.1 and −0.5 < log fold change (male/female) > 0.5. The average log fold change (male/female) is shown on the x axis. Enrichment for specific post-translational histone modifications was calculated for the 3 kb surrounding the TSS from ChIP-seq data generated from 2i ES cells (y axes). Correlations for sex dimorphic X-linked genes and TSS (**A**) H3K4me3 enrichment (t-test p-value < 2.2 × 10^−16^ (mean = −0.136)) (**B**) H3K9me3 enrichment (t-test p-value < 2.2 × 10^−16^ (mean = 0.619)) **(C)** H3K27me3 enrichment (t-test p-value = 0.04 (mean = 0.037)). Correlations for dimorphically expressed autosomal genes and TSS **(D)** H3K4me3 enrichment (ρ = 0.57, p-value < 2.2 × 10^−16^) (**E**) H3K9me3 enrichment (ρ = 0.0052, p-value = 0.89) (**F**) H3K27me3 enrichment (ρ = −0.077, p-value = 0.036).

Although power for *de novo* calling is limited by the number of biological replicates for the ES cells, we were able to identify sex-biased expression (genome-wide corrected p-value < 0.1, −0.5 < logFC > 0.5) for use in correlative analyses. Whilst the X-linked genes identified as sex-dimorphic in eight-cell embryos show evidence for escape from paternal X inactivation, they represent only a subset of the X-linked genes identified as sex-dimorphic in ES cells, where the paternally silenced X has been reactivated (Additional file 2: Figure S8). Consistent with what was found for the transcripts identified from eight-cell embryos, we observe that transcriptional activity of X-linked genes defined from ES cells is associated with enrichment of H3K4me3 in the 3 kb surrounding the TSS (Figure 4A; t-test p-value < 2.2x10^−16^ (mean = −0.136)). However, the ES cell defined X-linked dimorphic genes differ from the eight-cell embryo defined subset in that whilst male cells show H3K9me3 enrichment (Figure 4B; t-test p-value < 2.2x10^−16^ (mean = 0.619)), male specific H3K27me3 enrichment is very weak (Figure 4C; t-test p-value = 0.04 (mean = 0.037)), suggesting differential regulation of these gene subsets at the chromatin level, despite both maternal and paternally inherited alleles in both subsets being transcriptionally active in the ES cells.

A positive correlation between expression and H3K4me3 enrichment in the 3 kb surrounding the TSS of sex-dimorphic autosomal genes expressed in ES cells was found (Figure 4D; ρ = 0.57, p-value < 2.2x10^−16^). TSS H3K9me3 enrichment does not correlate with gene expression, although is enriched in males (Figure 4E; ρ = 0.0052, p-value = 0.89), whilst H3K27me3 is very weakly anti-correlated (Figure 4F; ρ = −0.077, p-value = 0.036). The autosomal genes identified in the eight-cell embryos were not called as significantly dimorphic in the ES cells, although they were still expressed. Furthermore, as there were so few sexually dimorphic autosomal genes in the eight-cell signature, we could not make correlations with the ESC histone marks. Our findings suggest that sex-biased gene expression associates with enrichment of H3K4me3 at the TSS in the sex that has higher expression. This relationship is true for both X-linked and autosomal genes.

### Sexual dimorphism is more pronounced in adult compared to fetal liver

Sexually dimorphic expression of the eight-cell signature genes in liver at adult and fetal stages did not recapitulate that found in pre-implantation development (Figure 2). To investigate the inter-relationship of sex-biased gene expression at later developmental stages, we identified a signature of 1488 dimorphic genes within adult liver (genome-wide corrected P < 0.1, −0.5 < logFC > 0.5; Additional file 3: Table S2). Autosomal genes account for ~96% of the dimorphic genes, in contrast to what is observed at pre-implantation stages (Additional file 2: Figure S8). KEGG analyses identified many enriched pathways, with drug metabolism as most significant, consistent with previous findings (Additional file 4: Table S3) [30]. Reassuringly, our data strongly correlated with previously published adult liver dimorphic differences, despite the different profiling platforms and genetic backgrounds between the two datasets (ρ = 0.82; p-value < 2.2x10^−16^) [24]. On the premise of this, we established a cut off (−0.5 < logFC > 0.5) by which we could define a signature for the other tissues; adipose, muscle and brain, profiled by Yang *et al*. Consistent with previous analyses we found that there was not a significant correlation for sex-dimorphic expression across tissues (data not shown), whilst X-linked genes showed modest female-biased expression differences [31]. Furthermore, the signature defined from eight-cell embryos did not show directional consistency in any of the adult tissues (data not shown).

**Figure 5:**
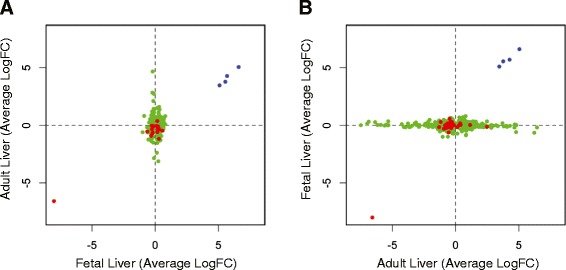
Sexually dimorphic expression across fetal and adult liver does not show directional consistency. Transcripts were defined as sex dimorphic from RNA-seq data using a genome-wide corrected p-value < 0.1 and −0.5 < log fold change (male/female) > 0.5. X-linked genes are shown in red, Y-linked genes are shown in blue and autosomal genes are shown in green (**A**) The average log fold change (male/female) for sex dimorphic genes defined from fetal liver (x axis) correlated with expression in the adult liver (y axis); X-linked (ρ = 0.094, p-value = 0.70), autosomal (ρ = 0.068, p-value = 0.19). (**B**) The log fold change (male/female) for sex dimorphic genes defined from adult liver (x axis) correlated with expression in the fetal liver (y axis) X-linked (ρ = 0.013, p-value = 0.93), autosomal (ρ = −0.042, p-value = 0.13).

We also defined a sexually dimorphic signature of 394 genes from late gestation fetal liver (genome-wide corrected p-value < 0.1, −0.5 < logFC > 0.5; Additional file 3 Table S2). As with adult liver, the majority (~94%) of sex-biased differences were autosomal in origin (Additional file 2: Figure S8). KEGG analysis identified 8 signaling and disease-associated pathways that were enriched in fetal liver after Bonferroni correction p-value < 0.05; (Additional file 4: Table S3). Using the same criteria, we identified 3.8X as many sex-dimorphic transcripts in adult compared to fetal liver. There was substantial overlap between the two developmental stages, with 72% (264/369) of autosomal, 67% (14/21) of X-linked and 100% (4/4) of Y-linked genes identified as dimorphic in fetal liver also being identified as dimorphic in adult liver (Additional file 3: Table S2), collectively representing a 11.95 fold enrichment (p-value < 0.001), when all expressed genes are considered as background. Yet, this enriched subset of dimorphic transcripts that is common across both fetal and adult liver do not show directional consistency across datasets (Figure 5) (X-squared = 2.07, p-value = 0.15). Our findings suggest that sexually dimorphic gene expression is present in late gestation liver, but is further expanded upon in the equivalent adult tissues, with some sexual-dimorphism being developmental stage specific. Consistent with previous findings, we did not find any up-regulation of X chromosome expression to equate with the level of autosomal gene expression, thereby refuting Ohno’s hypothesis [32]. The expression level of the X chromosome relative to autosomal genes was remarkably similar in males and females (1.84X and 1.82X, respectively for adult liver), but showed some variation according to developmental stage (1.43X and 1.43X, respectively for fetal liver).

Sex-based expression differences in adult liver are known to be largely dependent on differences in growth hormone regulation in response to sex-specific gonadal hormones [33,34]. To test if growth hormone responsiveness underlies the differences in dimorphic expression between fetal and adult liver, we asked if the dimorphic genes present in adult, but not fetal liver were enriched for previously identified growth hormone responsive transcripts [35]. Indeed, adult-specific sex-dimorphic genes had a 5.4X enrichment (p < 0.001). Collectively, our results suggest that sex-biased expression of some genes in liver is intrinsic, but that sex differences in growth hormone induces dimorphism of additional genes. Our data suggests that this latter mechanism is not yet operational in late gestation.

## Discussion

We present a whole-genome view of *in vivo* sexually dimorphic gene expression throughout mouse development, revealing that sex-specific expression biases in the embryo, fetus and adult are largely distinct.

Our study of dimorphism in pre-implantation development provides advances on previous work, by sampling prior to any cellular differentiation and eliminating culture induced artifacts [11,36,37]. By defining a high-confidence signature from eight-cell embryos we could then use this signature to probe other pre-implantation developmental stages despite the data being underpowered for defining sex dimorphic signatures *de novo*. We find that pre-implantation development is characterised by conserved dimorphism of sex-chromosome-linked genes, predominantly from the X chromosome [38]. Female-biased expression of some X-linked genes arises in two cell embryos, around the time embryonic genome activation is initiated in the mouse. Whether this is indicative of differences in the rate at which maternally inherited transcripts are degraded amongst sexes, or otherwise results from nascent transcription is unknown [14]. However, at least in the case of *Fthl17*, an X-linked maternally imprinted gene, transcription from the paternally inherited chromosome in the early embryo is responsible for female only expression [36]. Some of the X-linked genes in our eight-cell signature have been shown to escape paternal X inactivation in extra-embryonic tissues [39,40]. Allelic information suggests that for many of these genes, escape from paternal X silencing might also underlie their dimorphism in pre-implantation development. Consistent with previous findings, absolute paternal expression is either associated with the establishment of X inactivation (with the exception of *Fthl17*), whilst partial expression is more common amongst genes distal from the X inactivation centre [26] These genes also show a selective depletion of H3K27me3 in female ES cells, in contrast to sex-dimorphic X-linked genes that are not dimorphic in the eight cell embryos, even though the transcriptional behaviour between these subsets is similar in the ES cells. The difference between gene subsets escaping imprinted X inactivation in the pre-implantation embryo and placenta might be accounted for by progressive silencing of the paternal X chromosome throughout the pre-implantation period [27]. Similar X-linked dimorphism may not be conserved in humans, however, as regulation of X-inactivation is substantially different between species [41].

Autosomal genes also show dimorphism in pre-implantation stages, but sex-specific expression of these genes seems to be temporally restricted. Although we did not see conservation of these differences at other pre-implantation stages, we demonstrate validation in two independent cohorts of eight-cell embryos. The identification of dimorphic autosomal genes in eight-cell embryos implies regulation by sex-specific *trans* acting factors. In epiblast-like ES cells, autosomal genes highly expressed in one sex had corresponding enrichment for H3K4me3 around the TSS, consistent with what has been observed for strongly sex-biased genes in mouse liver [42]. Our eight-cell dimorphic signature included both X-linked transcription factors, e.g. *YY2*, and chromatin modifiers e.g. *Kdm6a* [37]. Interestingly, *Kdm6a*, has been shown to regulate specific targets, such as the *Rhox6/9* cluster in a sex-specific manner in ES cells [29], whilst its Y-encoded homolog (*Uty*), does not completely recapitulate its function [43,44]. Sex-chromosome complement might otherwise influence autosomal gene transcription through the inactive X chromosome influencing heterochromatic gene silencing in *trans* [45].

In line with previous findings, we found a large number of dimorphically expressed genes in adult liver that were not conserved across other adult tissues. Expanding on this, we also show that directionality was not conserved with the pre-implantation signature. We did, however, identify a subset of genes that show dimorphic expression in both fetal and adult liver. KEGG pathway analysis revealed dimorphism in common pathways relating to metabolism and stage-specific pathways relating to signal transduction in the liver at these two stages. Many additional genes were dimorphic in adults, possibly due to the activational effects of gonadal hormones. Consistent with this hypothesis we show that adult-specific liver dimorphic genes are enriched for growth hormone responsiveness [35]. Although gonadal hormones are produced in late gestation, the regulation of pituitary growth hormone secretion by gonadal hormones is minimal prior to puberty in mice [46].

## Conclusions

Our findings reveal that there are core transcriptional differences between the sexes that are consistent throughout pre-implantation development. Most of the genes identified in eight-cell embryos do not retain significantly dimorphic expression in fetal and adult tissues. Although sex-dimorphic expression is highly tissue-specific, a subset of genes is conserved across fetal and adult liver suggesting that sex-biased expression of this subset may be due to sex-chromosome complement, whilst differences are more likely driven by sex-specific physiology, which differs according to the stage of development. Although at a gene level we see a dramatic difference across pre-implantation, fetal and adult development, pathway level analysis reveals some conservation. By providing a genome-wide view of sex-dimorphic expression from post-fertilisation to adult, we hope to improve the understanding of the underlying molecular biology of sexually dimorphic phenotypes.

There is substantial evidence to suggest that sex-specific responses to environmental stimuli can occur prior to exposure to sex-specific hormones, and in some instances, even when the exposure is restricted to the previous generation [47]. Sex-chromosome complement can drive dramatic effects, as exemplified by the epigenome-wide differences in male and female murine ES cells when cultured in the presence of serum or defined medium [22,48]. Such effects may have lasting impact on developmental trajectories and disease risk [12]. Through characterising sexually dimorphic gene expression through a developmental trajectory, we reveal novel aspects of sex-specific biology and the inter-relationship of key phases across development. Our findings provide a platform for future work exploring the role of sex in moderating gene-environment interactions and highlight the importance of incorporating sex in studies of common disease and interventions [9,49].

## Methods

### Animal maintenance and tissue isolation

All animal procedures were conducted in accordance with the Home Office Animals (Scientific Procedures) Act 1986 (Project License number - 70/6693). C57BL/6 J mice were obtained from Charles River Laboratories, UK. All animals were maintained on standard laboratory chow and a 12 hr light/dark cycle. Male mice were housed with virgin females overnight. The detection of a vaginal plug the following morning was considered 0.5 dpc. Females were killed at 2.5 dpc and eight-cell embryos isolated from the fallopian tubes by flushing. Alternatively, females were killed at 17.5 dpc and embryonic liver was collected and snap frozen with liquid nitrogen. Adult animals were killed at 15–20 weeks of age and tissues collected and snap frozen. All animals were killed between 10 am and 12 pm.

### Single cell isolation from 8 cell embryos

Single embryos were transferred to acidic Tyrode’s solution to remove the zona pellucida, then washed in PBS-BSA (1 mg/mL) and dissociated into single cells as described previously [50]. All cells from a single embryo were used to generate a single RNA-seq library. For the discovery set, embryos of both sexes were derived from 4 independent litters, embryos used for biological replication were derived from an Additional 5 independent litters. The sires of the replicate litters were exposed to *in utero* protein restriction.

### Derivation of embryonic stem (ES) cell lines

ES cells were derived from eight-cell embryos as previously described [51]. Briefly, isolated eight-cell embryos were cultured in KSOM medium supplemented with (2i): mitogen-activated protein kinase inhibitor (PD0325901, 1 μM) and glycogen synthase kinase-3 inhibitor (CHIR99021, 3 μM). After two days, embryos were transferred to NDiff227 medium with 2i and LIF and allowed to develop into blastocysts. The trophectoderm was lysed by immunosurgery, and the ICM (inner cell mass) of each embryo plated in NDiff227 supplemented with 2i and LIF and for expansion to generate 2i ES cell lines.

### Generation and sequencing of RNA-seq libraries

The eight-cell embryo RNA-seq libraries were generated using a single-cell protocol adapted from [50]. Further details are provided in *Supplementary Information*.

### Generation and sequencing of ChIP-seq libraries

The chromatin immunoprecipitation (ChIP) assay was performed according to previously published protocols with minor modifications [52]. Chromatin was sonicated to get fragments of 100 to 700 bp and immunoprecipitated with the following antibodies anti-H3K27me3 (07–449, Millipore), anti-H3K4me3 (39159, Active Motif) and anti-H3K9me3 (ab8898, Abcam). ChIP-seq libraries were prepared using the Illumina ChIP-seq librarary prep kit, according to the manufacturers’ instructions.

### Analysis

All analysis was performed on UCSC reference genome mm10 and the gene annotation file downloaded from Tophat website (downloaded on September 25^th^ 2013).

### RNA-Seq analysis

All RNA-Seq data was mapped using Tophat v2.0.4 with Bowtie 2 v2.1.0 and samtools v0.1.18 using default settings. The mean insert sizes and standard deviations were calculated *in silico* using Picard Tools v1.98 from 1,000,000 reads. Duplicates were filtered using Picard Tools and reads were assigned to a gene using HTSeq. Differential analysis was performed using DESeq2 v1.4.5 which uses a generalised linear model in which counts are modelled using a negative binomial distribution. Genome wide corrected p-values were calculated using Benjamini-Hochberg multiple testing adjustment procedure. Normalised variance stabilizing transformed counts were used for all plots and further analysis. Existing public data was extracted as raw counts and analysed with DESeq2 in the same manner as our data.

### ChiP-Seq analysis

All ChiP-Seq data was mapped using Bowtie 2 v2.1.0 with default settings. Coverage across the genome was calculated using genomeCoverageBed from Bedtools v2.17.0 and converted to BigWig format using bedGraphToBigWig downloaded from the UCSC website. For correlation with RNA-Seq data the average coverage over +/−1.5 kbp of the TSS of each gene was calculated using bigWigAverageOverBed.

### Chromosome dosage model

To account for the difference in chromosome dosage between the sexes we defined a model based on the distribution of reads for the 2 male samples. We first removed the Y chromosome and then counted the (average) total number of reads (across the 2 samples) within a 3 kb window around the TSS of each of the genes across all remaining chromosomes including chrX (*N*_*total*_). We then calculated the average total number of reads on the X chromosome (*N*_*x*_). A scaled read density for chromosome X genes was then calculated:

$$ {S}_i=\frac{2{N}_i}{N_{total}+{N}_x} $$

Where *N*_*i*_ represents the number of reads for gene *i* on chromosome X. We can then compare this to the actual (average) read density for the X chromosome of the female samples calculated as:

$$ {A}_i=\frac{N_i}{N_{ftotal}} $$

where *N*_*ftotal*_ is the total number of reads (excluding the y-chromosome) for the female samples and *N*_*i*_ represents the number of reads for gene *i* on chromosome X.

### Data access

ChIP-seq data and RNA-seq data are available in the NCBI Gene Expression Omnibus (GSE59222), and will be made public upon acceptance of the manuscript for publication.

## Additional files

Additional file 1: Table S1. High-throughput data sequencing statistics. Attached as an Excel document. ChIP-seq and RNA-seq data statistics are provided on different worksheets.

Additional file 2: **Supplementary material.**

Additional file 3: Table S2. Genes identified as sexually dimorphic from adult and fetal liver. Sex dimorphic genes are defined from RNA-seq data derived from 12 female and 12 male samples in each set (adjusted p-value < 0.1, (Benjamini-Hochberg correction) and −0.5 < log(male/female) > 0.5). Dimorphic genes unique to fetal liver, common to both fetal and adult liver, or unique to adult liver are given on separate worksheets.

Additional file 4: Table S3. KEGG pathway analysis of sex dimorphic genes defined from fetal and adult liver. KEGG enrichment for sex-dimorphic genes unique to fetal liver, common to both fetal and adult liver, or unique to adult liver are given on separate worksheets. All genes with detectable expression were used as background for enrichment analysis.

## References

[1] Williams TM, Carroll SB (2009). Genetic and molecular insights into the development and evolution of sexual dimorphism. Nat Rev Genet.

[2] Yang F, Babak T, Shendure J, Disteche CM (2010). Global survey of escape from X inactivation by RNA-sequencing in mouse. Genome Res.

[3] Ober C, Loisel DA, Gilad Y (2008). Sex-specific genetic architecture of human disease. Nat Rev Genet.

[4] Dimas AS, Nica AC, Montgomery SB, Stranger BE, Raj T, Buil A (2012). Sex-biased genetic effects on gene regulation in humans. Genome Res.

[5] Liu LY, Schaub MA, Sirota M, Butte AJ (2012). Sex differences in disease risk from reported genome-wide association study findings. Hum Genet.

[6] De Vries GJ, Rissman EF, Simerly RB, Yang LY, Scordalakes EM, Auger CJ (2002). A model system for study of sex chromosome effects on sexually dimorphic neural and behavioral traits. J Neurosci.

[7] Chen X, McClusky R, Itoh Y, Reue K, Arnold AP (2013). X and Y chromosome complement influence adiposity and metabolism in mice. Endocrinology.

[8] Kuljis DA, Loh DH, Truong D, Vosko AM, Ong ML, McClusky R (2013). Gonadal- and sex-chromosome-dependent sex differences in the circadian system. Endocrinology.

[9] Aiken CE, Ozanne SE (2013). Sex differences in developmental programming models. Reproduction.

[10] Kobayashi S, Isotani A, Mise N, Yamamoto M, Fujihara Y, Kaseda K (2006). Comparison of gene expression in male and female mouse blastocysts revealed imprinting of the X-linked gene, Rhox5/Pem, at preimplantation stages. Curr Biol.

[11] Bermejo-Alvarez P, Rizos D, Rath D, Lonergan P, Gutierrez-Adan A (2010). Sex determines the expression level of one third of the actively expressed genes in bovine blastocysts. Proc Natl Acad Sci U S A.

[12] Donjacour A, Liu X, Lin W, Simbulan R, Rinaudo P (2014). In Vitro Fertilization Affects Growth and Glucose Metabolism in a Sex-Specific Manner in an Outbred Mouse Model. Biol Reprod.

[13] Wang H, Dey SK (2006). Roadmap to embryo implantation: clues from mouse models. Nat Rev Genet.

[14] Deng Q, Ramskold D, Reinius B, Sandberg R (2014). Single-cell RNA-seq reveals dynamic, random monoallelic gene expression in mammalian cells. Science.

[15] Park SJ, Komata M, Inoue F, Yamada K, Nakai K, Ohsugi M (2013). Inferring the choreography of parental genomes during fertilization from ultralarge-scale whole-transcriptome analysis. Genes Dev.

[16] Chureau C, Chantalat S, Romito A, Galvani A, Duret L, Avner P (2011). Ftx is a non-coding RNA which affects Xist expression and chromatin structure within the X-inactivation center region. Hum Mol Genet.

[17] Hemberger M, Kurz H, Orth A, Otto S, Luttges A, Elliott R (2001). Genetic and developmental analysis of X-inactivation in interspecific hybrid mice suggests a role for the Y chromosome in placental dysplasia. Genetics.

[18] de Waal E, Yamazaki Y, Ingale P, Bartolomei MS, Yanagimachi R, McCarrey JR (2012). Gonadotropin stimulation contributes to an increased incidence of epimutations in ICSI-derived mice. Hum Mol Genet.

[19] Ying QL, Wray J, Nichols J, Batlle-Morera L, Doble B, Woodgett J (2008). The ground state of embryonic stem cell self-renewal. Nature.

[20] Marks H, Kalkan T, Menafra R, Denissov S, Jones K, Hofemeister H (2012). The transcriptional and epigenomic foundations of ground state pluripotency. Cell.

[21] Leitch HG, McEwen KR, Turp A, Encheva V, Carroll T, Grabole N (2013). Naive pluripotency is associated with global DNA hypomethylation. Nat Struct Mol Biol.

[22] Habibi E, Brinkman AB, Arand J, Kroeze LI, Kerstens HH, Matarese F (2013). Whole-genome bisulfite sequencing of two distinct interconvertible DNA methylomes of mouse embryonic stem cells. Cell Stem Cell.

[23] Biase FH, Cao X, Zhong S (2014). Cell fate inclination within 2-cell and 4-cell mouse embryos revealed by single-cell RNA sequencing. Genome Res.

[24] Yang X, Schadt EE, Wang S, Wang H, Arnold AP, Ingram-Drake L (2006). Tissue-specific expression and regulation of sexually dimorphic genes in mice. Genome Res.

[25] Okamoto I, Otte AP, Allis CD, Reinberg D, Heard E (2004). Epigenetic dynamics of imprinted X inactivation during early mouse development. Science.

[26] Huynh KD, Lee JT (2003). Inheritance of a pre-inactivated paternal X chromosome in early mouse embryos. Nature.

[27] Patrat C, Okamoto I, Diabangouaya P, Vialon V, Le Baccon P, Chow J (2009). Dynamic changes in paternal X-chromosome activity during imprinted X-chromosome inactivation in mice. Proc Natl Acad Sci U S A.

[28] Reichmann J, Reddington JP, Best D, Read D, Ollinger R, Meehan RR (2013). The genome-defence gene Tex19.1 suppresses LINE-1 retrotransposons in the placenta and prevents intra-uterine growth retardation in mice. Hum Mol Genet.

[29] Berletch JB, Deng X, Nguyen DK, Disteche CM (2013). Female bias in Rhox6 and 9 regulation by the histone demethylase KDM6A. PLoS Genet.

[30] Waxman DJ, Holloway MG (2009). Sex differences in the expression of hepatic drug metabolizing enzymes. Mol Pharmacol.

[31] Reinius B, Johansson MM, Radomska KJ, Morrow EH, Pandey GK, Kanduri C (2012). Abundance of female-biased and paucity of male-biased somatically expressed genes on the mouse X-chromosome. BMC Genomics.

[32] Xiong Y, Chen X, Chen Z, Wang X, Shi S, Wang X (2010). RNA sequencing shows no dosage compensation of the active X-chromosome. Nat Genet.

[33] van Nas A, Guhathakurta D, Wang SS, Yehya N, Horvath S, Zhang B (2009). Elucidating the role of gonadal hormones in sexually dimorphic gene coexpression networks. Endocrinology.

[34] Zhang Y, Laz EV, Waxman DJ (2012). Dynamic, sex-differential STAT5 and BCL6 binding to sex-biased, growth hormone-regulated genes in adult mouse liver. Mol Cell Biol.

[35] Wauthier V, Sugathan A, Meyer RD, Dombkowski AA, Waxman DJ (2010). Intrinsic sex differences in the early growth hormone responsiveness of sex-specific genes in mouse liver. Mol Endocrinol.

[36] Kobayashi S, Fujihara Y, Mise N, Kaseda K, Abe K, Ishino F (2010). The X-linked imprinted gene family Fthl17 shows predominantly female expression following the two-cell stage in mouse embryos. Nucleic Acids Res.

[37] Chen L, Wang D, Wu Z, Ma L, Daley GQ (2010). Molecular basis of the first cell fate determination in mouse embryogenesis. Cell Res.

[38] Rinn JL, Snyder M (2005). Sexual dimorphism in mammalian gene expression. Trends Genet.

[39] Finn EH, Smith CL, Rodriguez J, Sidow A, Baker JC (2014). Maternal bias and escape from X chromosome imprinting in the midgestation mouse placenta. Dev Biol.

[40] Garrick D, Sharpe JA, Arkell R, Dobbie L, Smith AJ, Wood WG (2006). Loss of Atrx affects trophoblast development and the pattern of X-inactivation in extraembryonic tissues. PLoS Genet.

[41] Okamoto I, Patrat C, Thepot D, Peynot N, Fauque P, Daniel N (2011). Eutherian mammals use diverse strategies to initiate X-chromosome inactivation during development. Nature.

[42] Sugathan A, Waxman DJ (2013). Genome-wide analysis of chromatin states reveals distinct mechanisms of sex-dependent gene regulation in male and female mouse liver. Mol Cell Biol.

[43] Shpargel KB, Sengoku T, Yokoyama S, Magnuson T (2012). UTX and UTY demonstrate histone demethylase-independent function in mouse embryonic development. PLoS Genet.

[44] Welstead GG, Creyghton MP, Bilodeau S, Cheng AW, Markoulaki S, Young RA (2012). X-linked H3K27me3 demethylase Utx is required for embryonic development in a sex-specific manner. Proc Natl Acad Sci U S A.

[45] Wijchers PJ, Yandim C, Panousopoulou E, Ahmad M, Harker N, Saveliev A (2010). Sexual dimorphism in mammalian autosomal gene regulation is determined not only by Sry but by sex chromosome complement as well. Dev Cell.

[46] Conforto TL, Waxman DJ (2012). Sex-specific mouse liver gene expression: genome-wide analysis of developmental changes from pre-pubertal period to young adulthood. Biol Sex Differ.

[47] Ng SF, Lin RC, Laybutt DR, Barres R, Owens JA, Morris MJ (2010). Chronic high-fat diet in fathers programs beta-cell dysfunction in female rat offspring. Nature.

[48] Zvetkova I, Apedaile A, Ramsahoye B, Mermoud JE, Crompton LA, John R (2005). Global hypomethylation of the genome in XX embryonic stem cells. Nat Genet.

[49] Lucas E (2013). Epigenetic effects on the embryo as a result of periconceptional environment and assisted reproduction technology. Reprod Biomed Online.

[50] Tang F, Barbacioru C, Nordman E, Li B, Xu N, Bashkirov VI (2010). RNA-Seq analysis to capture the transcriptome landscape of a single cell. Nat Protoc.

[51] Nichols J, Silva J, Roode M, Smith A (2009). Suppression of Erk signalling promotes ground state pluripotency in the mouse embryo. Development.

[52] Cuddapah S, Jothi R, Schones DE, Roh TY, Cui K, Zhao K (2009). Global analysis of the insulator binding protein CTCF in chromatin barrier regions reveals demarcation of active and repressive domains. Genome Res.

