# Supplementary material for: Sexually dimorphic gene expression emerges with embryonic genome activation and is dynamic throughout development
Source: BMC Genomics. 2015 Apr 14;16(1):295. doi: 10.1186/s12864-015-1506-4 (PMC4410000; doi:10.1186/s12864-015-1506-4)
Supplement: Supplementary file 2 — Supplementary material. [file 12864_2015_1506_MOESM2_ESM.docx]

**Supplementary methods**

**Adapted single cell RNA-seq protocol**

Cell lysis, first and second strand synthesis were performed as described previously [1], with reaction volumes adjusted accordingly. The number of cycles for the first round of PCR amplification was reduced from 20 to 15. Replicate reactions were pooled. A 1:10 dilution was then used as template for qRT-PCR to assess the presence of two ubiquitously expressed transcripts, *Mon2* and *Fbxo38*. The remaining reaction was purified using the Qiagen QIAquick PCR purification kit and eluted in 30 μl. The size distribution was analysed on an Agilent Bioanalyzer using The High Sensitivity DNA Kit. A second round of amplification was performed using amine-blocked primers as described in [1]. For each library, 4 x 90 μl reactions, each with 100 pg of first round product were amplified for 9 cycles. Replicate reactions were pooled and cleaned up with 2 rounds of 0.5x volumes of AgenCourt Ampure XP beads, according to the manufacturers’ instructions. The products were again checked on the Bioanalyzer using The High Sensitivity DNA Kit to ensure sufficient removal of excess primers. 100 ng of the second round product was diluted to 130 μl in 10 mM Tris, pH7.5 and sheared using 4 cycles on the covaris (bath temperature 5^0^C, duty cycle 10%, intensity 5, 200 cycles/burst, 60 s). Sheared cDNA was size selected with 1.2 x vol of AgenCourt Ampure XP beads. 10 ng of this sheared cDNA was used as input for library preparation using the Illumina TruSeq ChIP Sample Preparation Kit. The protocol was adjusted, with a double size-selection being performed with AgenCourt Ampure XP beads instead of size selection from an agarose gel and PCR enrichment reduced from 18 to 8 cycles of amplification. Each library was indexed and pooled 12-plex for sequencing on a single lane of an Illumina Hi-Seq 2000.

RNA-seq libraries from embryonic stem cells, fetal and adult liver samples were constructed using the Illumina TruSeq Stranded Total RNA Kit, with 1 μg of Total RNA extracted with Qiagen miRNeasy Micro Kit. RNA integrity was assessed using an Agilent Bioanalyzer and considered acceptable with a RNA Integrity Number >8.5. The sex of embryonic stem cell lines and fetal samples was determined using a SRY-specific multiplex PCR targeting genomic DNA extracted concurrently, as described previously [2]. Samples were individually indexed and then pooled to 12-plex for sequencing on a single Hi-Seq 2000 lane.

**Supplementary figures**


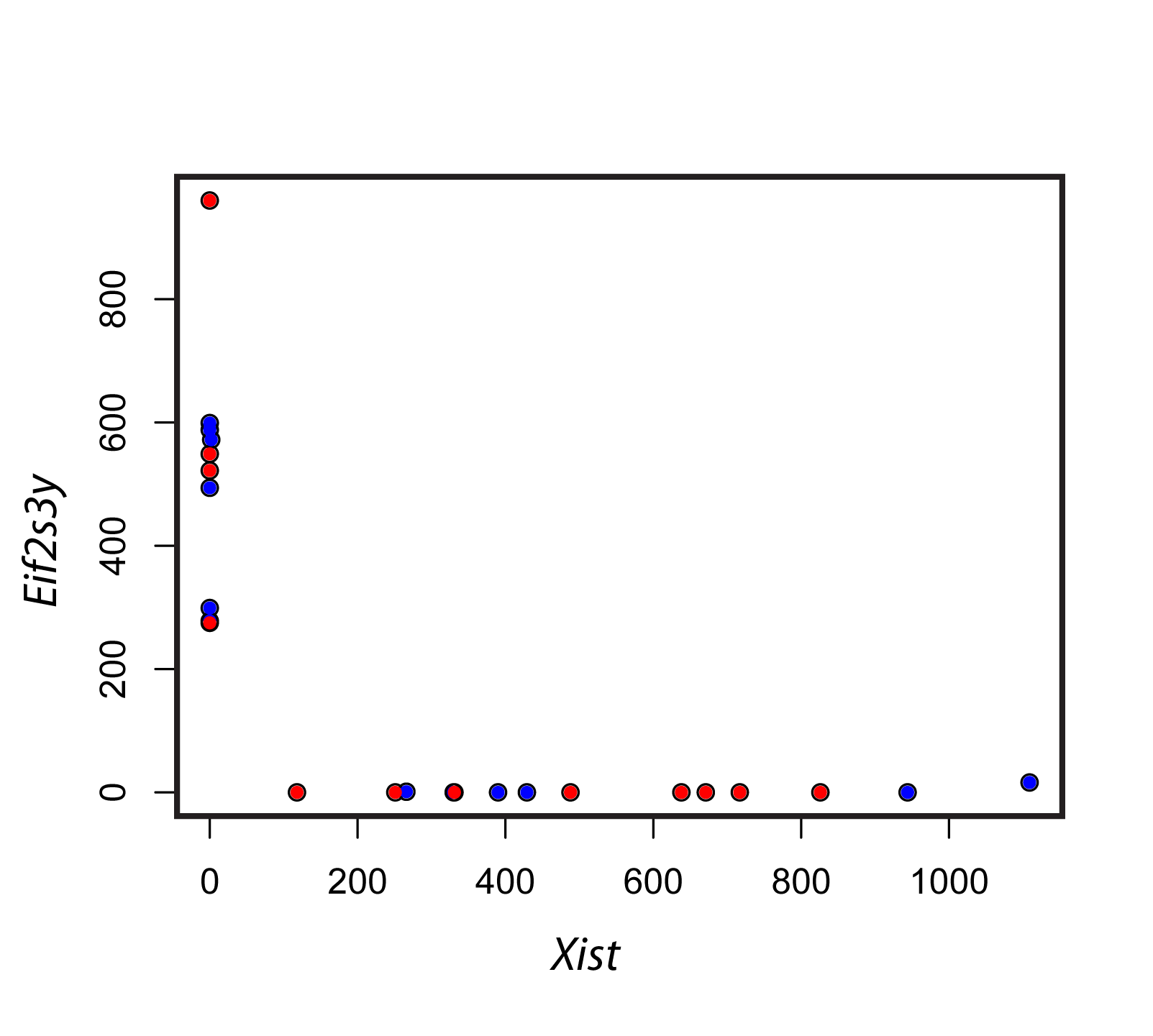


**Figure S1. Eight cell embryo sex determination**. Raw read counts for X-linked *Xist* and Y-linked *Eif2s3y* separates eight cell-embryos according to sex. The discovery set is indicated in blue, validation set is indicated in red.


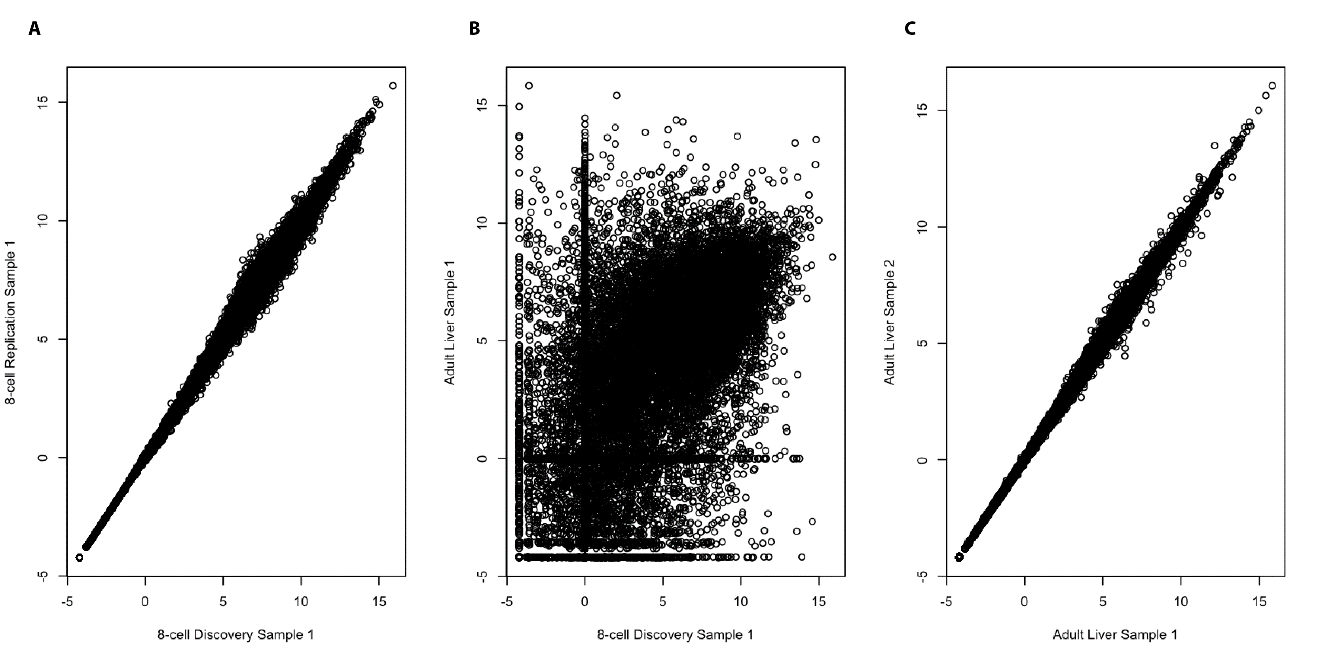


**Figure S2. Low technical (within group) variation in global gene expression profiles generated from eight cell embryos or adult liver of the same sex regardless of RNA-seq protocol**. Normalised read counts for **A)** All detected genes in a sample from the discovery group of eight cell embryos (x-axis), vs a sample from the replication experiment (y-axis) (ρ=0.998, p-value < 2.2x10^-16^). **B)** Biological variation between global expression patterns in eight cell embryo (x-axis) vs adult liver (y-axis) (ρ=0.61, p-value < 2.2x10^-16^) **C)** Technical variation within adult liver using global expression profiles (ρ=0.999, p-value < 2.2x10^-16^).


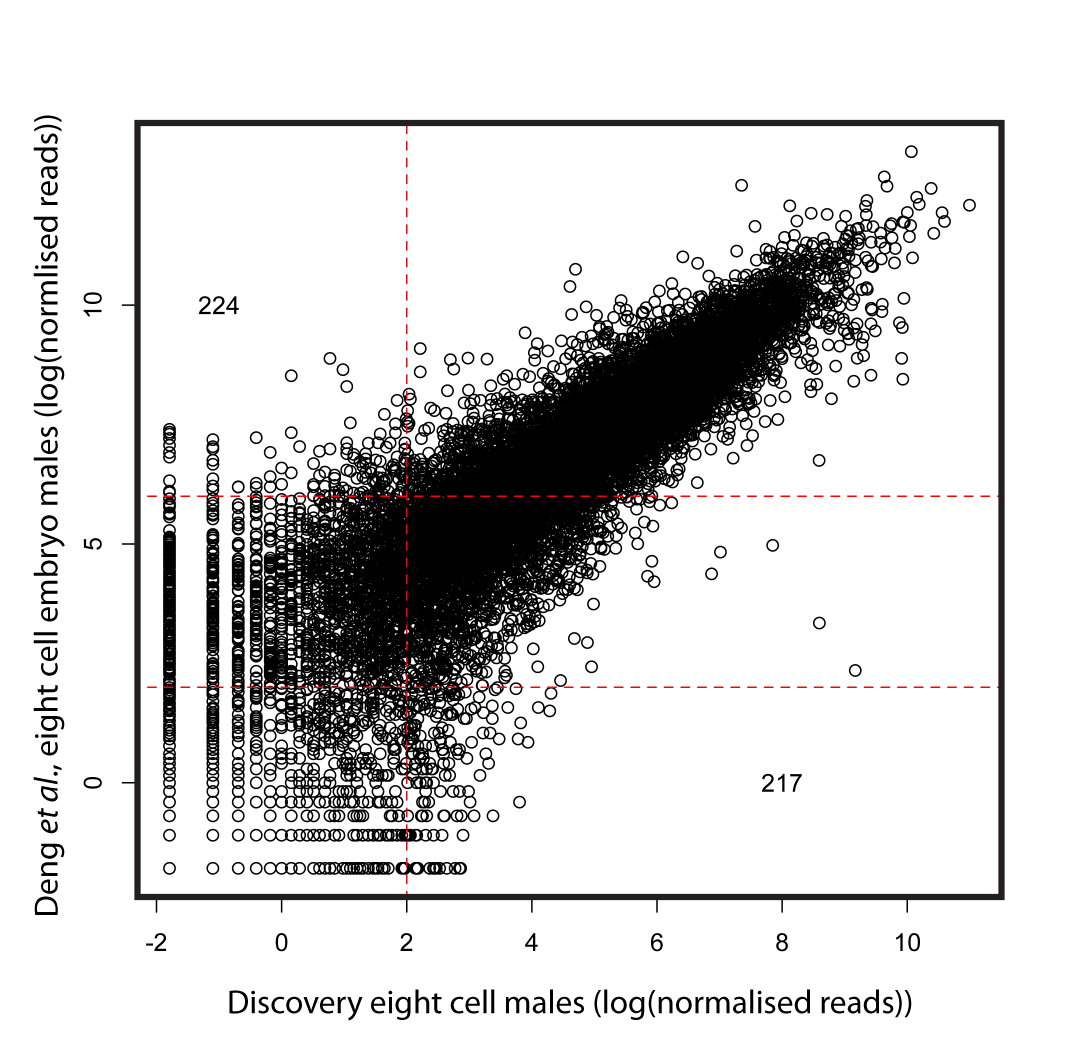


**Figure S3. Strong correlation of gene expression in discovery eight cell male embryos with those profiled by Deng *et al*.** Normalised log transformed expression patterns are plotted for all expressed genes (ρ =0.82, p-value < 2.2 x10^-16^).


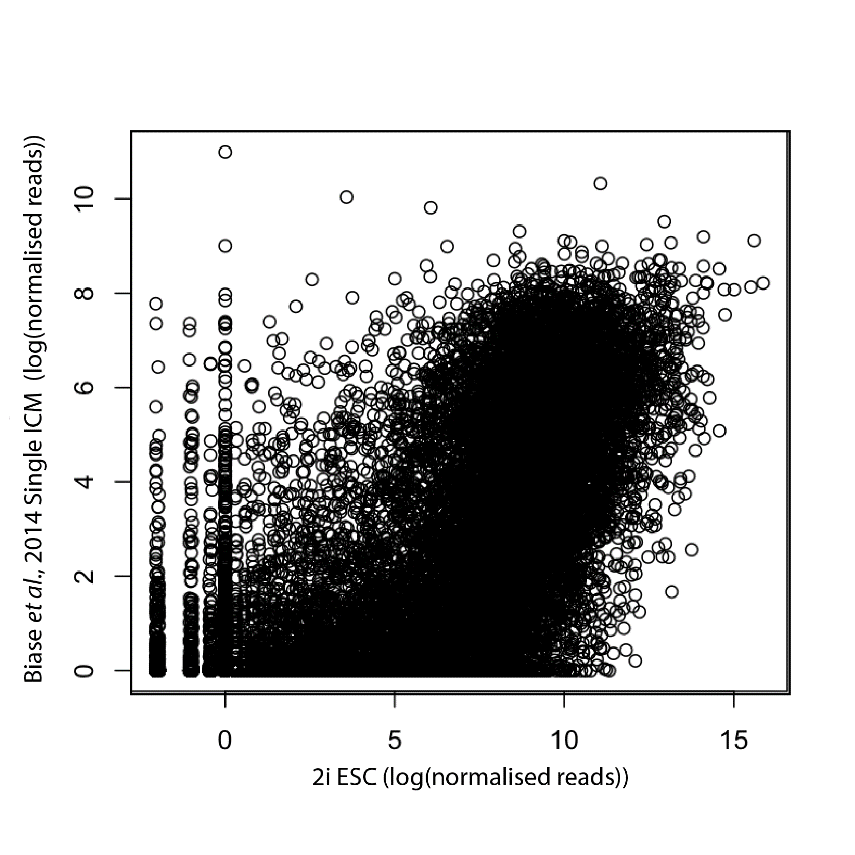


**Figure S4.** **Strong correlation of gene expression in discovery eight cell male embryos with those profiled by Biase *et al*.** Normalised log transformed expression patterns are plotted for all expressed genes (ρ =0.70, p-value < 2.2x10^-16^).


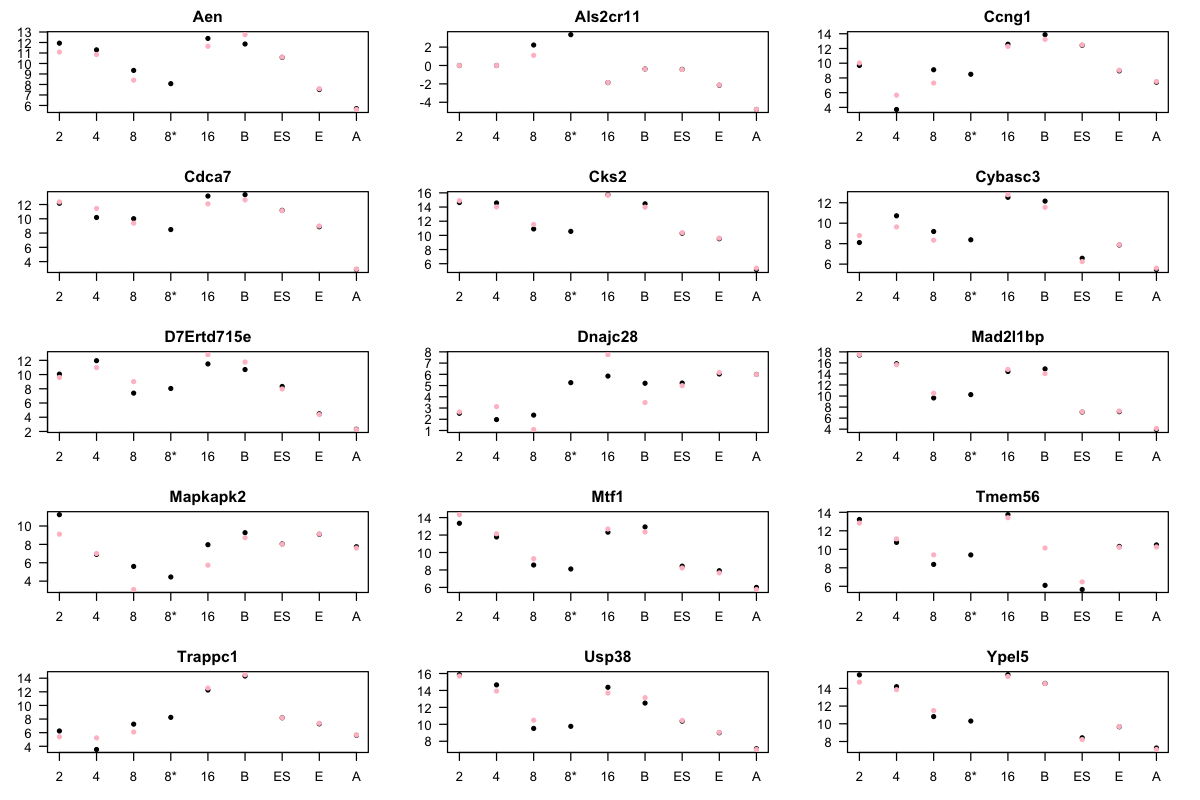


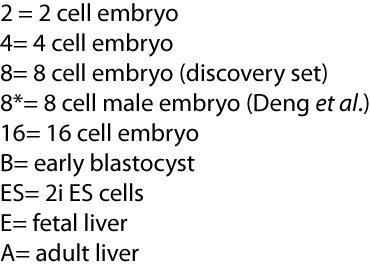


**Figure S5. Gene expression of sexually dimorphic autosomal genes defined from eight cell embryos is highly dynamic.** Gene names are indicated above each plot. Log normalised counts are plotted on the Y axis. Black points indicate expression levels in males. Pink points indicate expression levels in females.


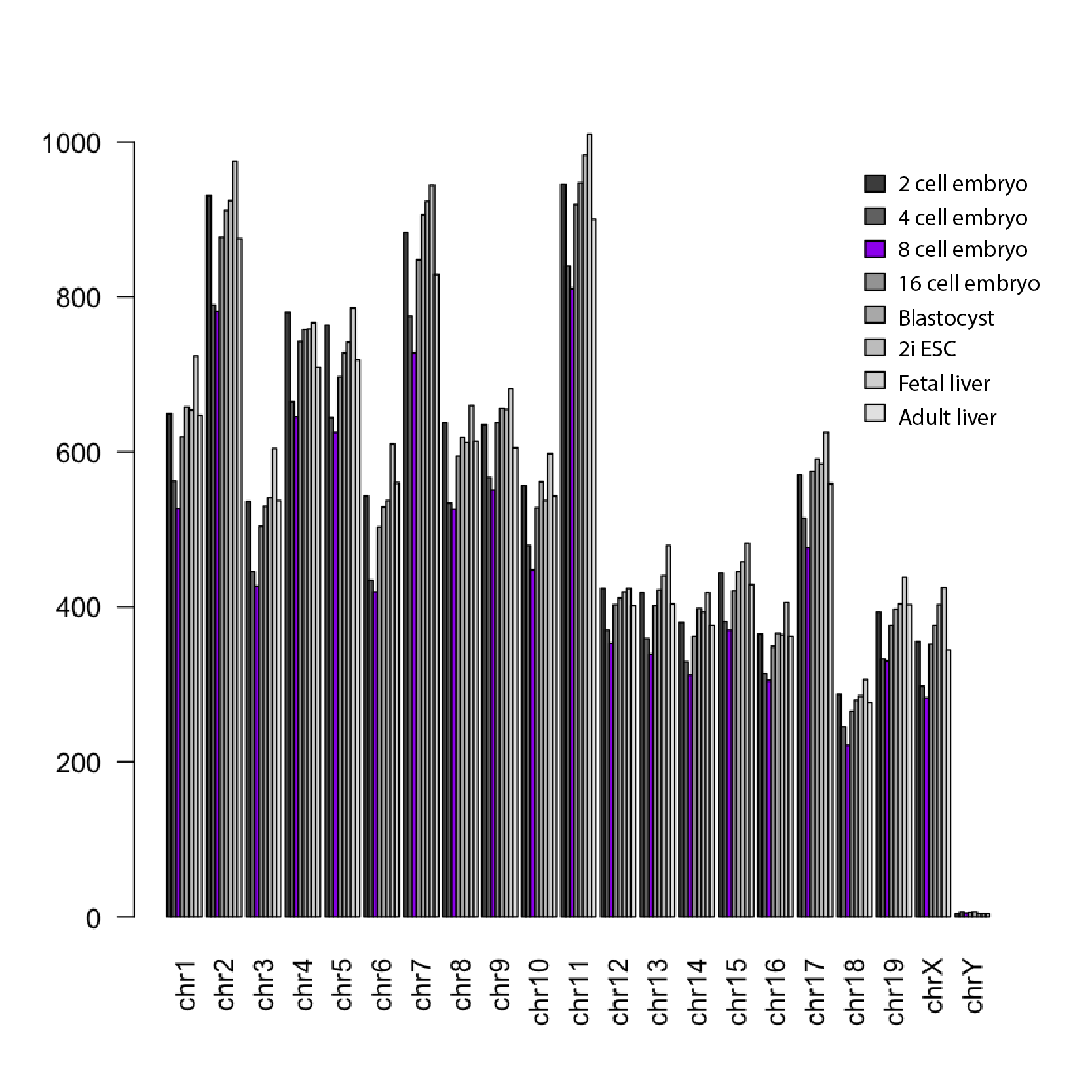


**Figure S6. The number of expressed transcripts is lowest in eight cell embryos compared to other developmental stages.** The total number of genes from each chromosome above detection threshold after normalisation is plotted.


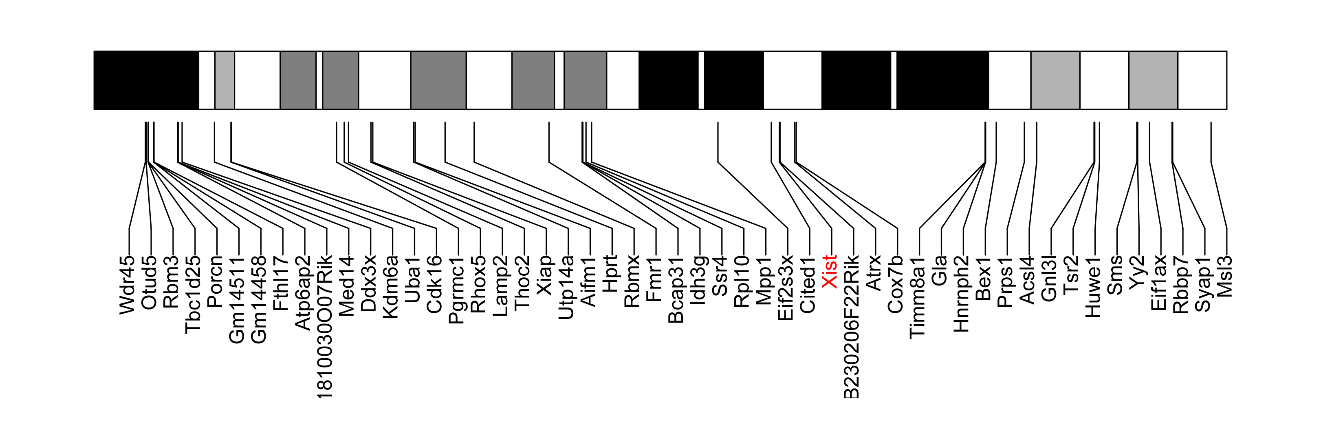


**Figure S7. An ideogram showing the chromosomal location of sexually dimorphic X-linked genes.** Most dimorphic genes are located distally from the X inactivation centre (indicated by the location of *Xist* (red)).


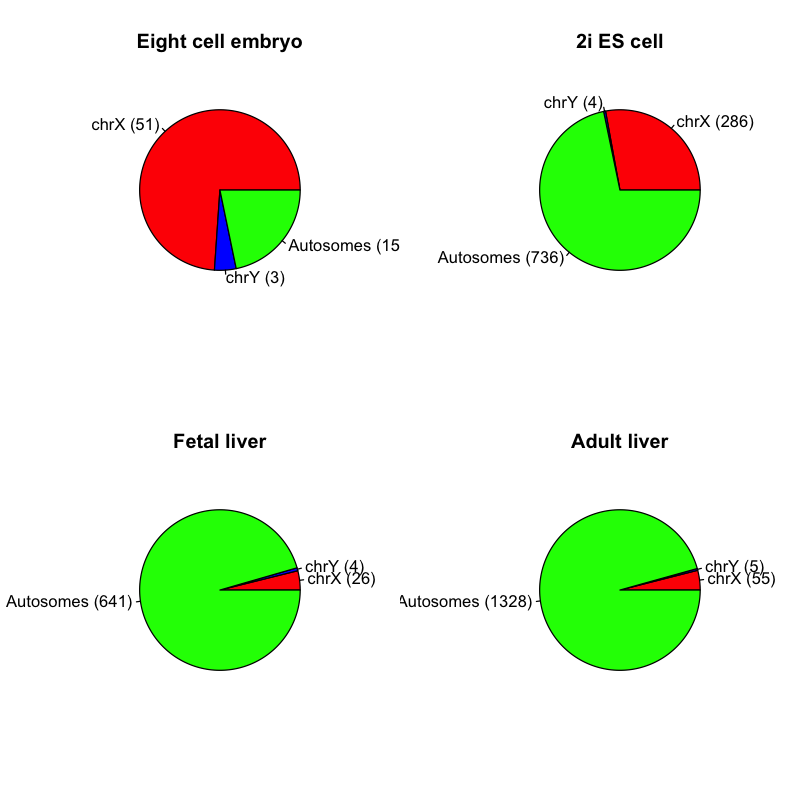


**Figure S8. Chromosomal location of genes defined as sexually dimorphic.** The pie chart show the contribution of Y-chromosome genes (blue), X chromosome genes (red) and autosomal genes (green) to the sexually dimorphic signatures defined from RNA-seq data at a genome-wide corrected p-value<0.1, and -0.5<log fold change (male/female)>0.5.


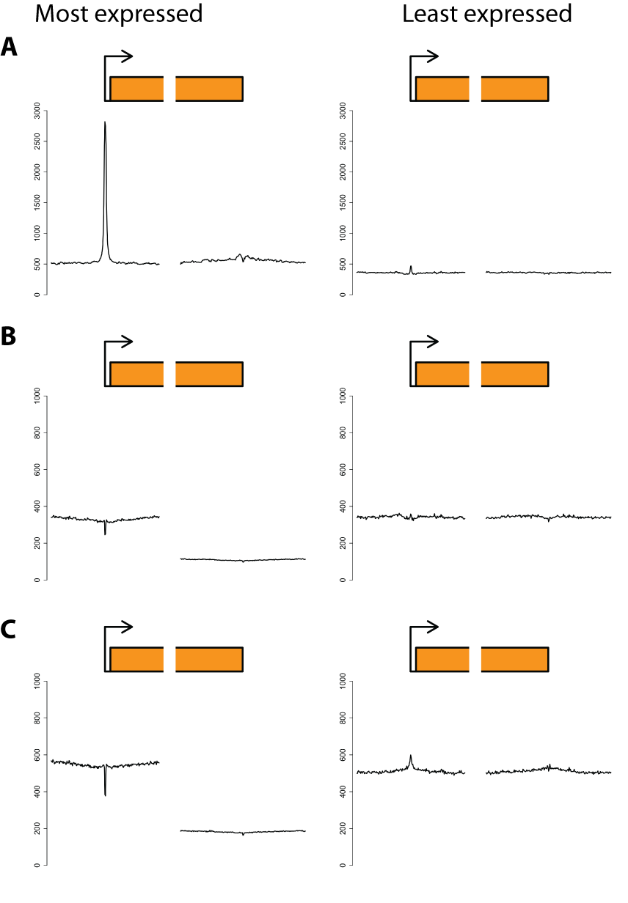


**Figure S9. Average profiles of post-translational histone marks for the most high or low-expressed genes in 2i ES cells confirm the expected distributions for ground state pluripotency.** Average profiles were derived for the 2000 most-(left) and 2000 least (right) expressed genes in our 2i ES cells. The coverage across the TSS and the 3’ end of each gene was calculated in a window of 100 kbp (+/- 50kbp). The window was segmented into regions of 500 bp and the total number of reads overlapping each region was calculated for each gene. (A)H3K4me3, (B) H3K9me3 and (C)H3K27me3.

**Supplementary references**

1. Tang F, Barbacioru C, Nordman E, Li B, Xu N, Bashkirov VI, Lao K, Surani MA: **RNA-Seq analysis to capture the transcriptome landscape of a single cell**. *Nat Protoc* 2010, **5**(3):516-535.

2. Davies TJ, Fairchild PJ: **Optimization of protocols for derivation of mouse embryonic stem cell lines from refractory strains, including the non obese diabetic mouse**. *Stem Cells Dev* 2012, **21**(10):1688-1700.

3. Biase FH, Cao X, Zhong S: **Cell fate inclination within 2-cell and 4-cell mouse embryos revealed by single-cell RNA sequencing.** *Genome Res* 2014, **24**(11):1787-96.
